# Addressing the Needs of Chinese American Dementia Caregivers in Feeding Decisions Through Community Engagement

**DOI:** 10.1093/geroni/igaf122.1416

**Published:** 2025-12-31

**Authors:** Yaolin Pei, Bei Wu, Terri Fried, Dena Schulman-Green, Laura Hanson

**Affiliations:** The University of Texas at Austin, Austin, Texas, United States; NYU Shanghai, Shanghai, China (People’s Republic); Yale University School of Medicine, New Haven, Connecticut, United States; New York University, New York, New York, United States; The University of North Carolina at Chapel Hill School of Medicine, Chapel Hill, North Carolina, United States

## Abstract

Feeding difficulties are common among individuals with advanced dementia. Dementia family caregivers have to make difficult decisions about feeding options. Despite limited clinical benefit, over 50% of Chinese individuals with advanced dementia receiving tube-feeding. Little is known about Chinese American dementia caregivers’ attitudes towards tube feeding and their decision-making needs regarding feeding options. In collaboration with CaringKind, a community agency serving dementia caregivers in NYC, we conducted a multi-method study among Chinese American dementia caregivers consisting of an original survey to capture knowledge/attitudes about feeding decisions and a qualitative descriptive study to inform a culturally adapted decision aid. We calculated descriptive statistics for quantitative data and used thematic analysis for qualitative data. The survey (n = 63) showed that participants had limited knowledge about tube feeding; 35% opted for tube feeding in an end-of-life scenario, while 41% were uncertain. Qualitative interviews with 21 Chinese American dementia caregivers and 13 healthcare providers indicated that: 1) caregivers value family-based and harmony-oriented decision-making and often misunderstand that careful hand feeding equates to starvation; and 2) Chinese American persons with dementia prefer indirect communication regarding their end-of-life care wishes. Based on study results, we culturally adapted an appropriate decision aid titled “Making choices: feeding options for patients with dementia” successfully used with dementia caregivers. We are collecting feedback from caregivers and health care providers to make refinements. Next, we will pilot test the feasibility, acceptability, and preliminary efficacy of the culturally adapted decision aid intervention to better address the needs of our caregiver group.

